# A Qualitative Exploration of the Perceptions of Women Living with Pelvic Floor Disorders and Factors Related to Quality of Life

**DOI:** 10.3390/jcm13071896

**Published:** 2024-03-25

**Authors:** Julián Rodríguez-Almagro, Antonio Hernández Martínez, Sergio Martínez-Vázquez, Rocío Adriana Peinado Molina, Alberto Bermejo-Cantarero, Juan Miguel Martínez-Galiano

**Affiliations:** 1 Department of Nursing, Physiotherapy and Occupational Therapy, Ciudad Real Faculty of Nursing, University of Castilla-La Mancha, 13001 Ciudad Real, Spain; antomatron@gmail.com (A.H.M.); alberto.bermejo@uclm.es (A.B.-C.); 2 Department of Nursing, University of Jaen, 23071 Jaen, Spain; svazquez@ujaen.es (S.M.-V.); rpmolina@ujaen.es (R.A.P.M.); jgaliano@ujaen.es (J.M.M.-G.); 3 Consortium for Biomedical Research in Epidemiology and Public Health (CIBERESP), 28029 Madrid, Spain

**Keywords:** pelvic floor disorders, perceptions, sexual dysfunction, women, qualitative, quality of life

## Abstract

**Background:** Pelvic floor dysfunction encompasses conditions like urinary and fecal incontinence, pelvic organ prolapse, and pelvic pain, significantly affecting women’s quality of life. Despite its prevalence, few studies have adopted a qualitative approach to understanding women’s perceptions and emotions regarding these issues. This study aims to delve into how women with pelvic floor disorders perceive their condition and its impact on their daily lives. **Methods:** We analyzed qualitative data from interviews with 160 women suffering from pelvic floor dysfunctions. Using inductive qualitative content analysis, we systematically examined the data to identify variations, differences, and similarities. **Results:** The analysis revealed four primary themes in the women’s narratives: “Physical Impacts”, “Emotional and Psychological Impacts”, “Social and Relational Impacts”, and “Sexual Health Impacts”, along with 12 subthemes. The findings predominantly highlight how pelvic floor dysfunctions detrimentally affect women’s quality of life and emotional well-being, instilling fear and insecurity in daily activities, compounded by sleep disturbances and sexual dysfunction. **Conclusions:** Women living with pelvic floor dysfunction face multifaceted challenges that adversely affect various aspects of their lives, diminishing their overall quality of life. This includes notable impacts on sleep, physical, and sexual activities. However, not all affected women report these issues, often due to fear of stigma, choosing instead to conceal their struggles in an effort to maintain an appearance of normalcy.

## 1. Introduction

Pelvic floor disorders (PFDs) are an important, highly prevalent problem that can potentially affect up to a third of adult women in countries like the United States [1] (espite the high prevalence, it is considered to be an underdiagnosed problem. An estimated 25% of patients consult a doctor for the condition, and this low percentage is attributed to embarrassment, a lack of health education, and scarcity of specific consultations that address the problem [2].

Pelvic floor dysfunction encompasses a series of problems that are common to the condition of this muscular structure, including urinary incontinence, pelvic organ prolapse in women, fecal incontinence, pelvic-perineal regional pain syndrome, and sexual dysfunction due to weak musculature in this region, among others [1]. 

Pelvic floor dysfunction is a serious health problem that negatively impacts women’s quality of life, affecting their physical activity, daily rest, and sexual activity, as well as causing changes in their psychological state [3,4,5,6,7,8,9,10]. 

Research on pelvic floor dysfunction in women has been primarily conducted with a quantitative approach and has largely focused on therapeutic management and prevalence studies, with less emphasis on the experiences of women living with and seeking care for this condition [11]. On the other hand, the few studies published with a qualitative approach on the experiences of women with pelvic organ dysfunction have focused on very specific aspects, such as the discomfort of the symptoms and their impact on daily life [6,12,13], sexual health and body image [14], seeking care for prolapse [15], and women’s perspectives on available treatment options [15].

Considering the above, our objective was to comprehensively and holistically explore the perceptions and emotions of women with pelvic floor dysfunction and how their daily lives are affected.

## 2. Methods

### 2.1. Ethical Approval

All authors participated in the validation of the results, questioning each step of the analysis to check any alternative interpretations. The analysis was discussed until an agreement was reached. Three researchers participated in the thematic analysis process to ensure consistency in the analysis and findings. 

This study has received the approval of the Research Ethics Committee of the province of Jaén (SPCV-0220/0320-N-20, 26 March 2020). Informed consent was obtained from all participants. All methods were performed in accordance with the relevant guidelines and regulations.

### 2.2. Study Design and Participants

We conducted a qualitative descriptive study [16] through inductive qualitative content analysis [17] using social networks for participant recruitment. An empirical phenomenological approach was used to obtain detailed descriptions of women’s experiences with pelvic floor problems and to know what spheres of their daily life were affected by it. The research team designed the questions (Appendix A) based on their clinical experiences. Participants were encouraged to write about their pelvic floor issues and how they affected their daily lives, answering in their own words.

First, the objectives of this study and its voluntary nature were explained to all the participants, and their informed consent was obtained before starting each questionnaire. Confidentiality was ensured by using numbers instead of names. Throughout the study, we followed the COREQ Standards for Reporting Qualitative Research guidelines [18]

The participants were recruited through purposive and snowball sampling [19]. The sample size was determined through the Saturation principle, i.e., the point at which no new themes appear, even though there are new participants [20]. All participants were women over 18 years of age with pelvic floor problems (urinary and fecal incontinence, pelvic organ prolapse, and pelvic pain). 

All participants were recruited through associations focused on pelvic floor disorders, as previously mentioned, which require a medical diagnosis for membership eligibility; this criterion ensures that all women involved in our study had been diagnosed with a pelvic floor condition. A total of 160 women responded to the questions asked. The following exclusion criteria were used: women with difficulty understanding Spanish (language barrier), who had given birth in the previous 12 months, and those with psychiatric and/or cognitive problems that could affect data collection.

During the process, the criteria for methodological rigor of credibility, dependability, confirmability, and transferability were observed [16]. All the authors participated in the validation of the results, questioning each step in the analysis to verify any possible alternative interpretations. The analysis was discussed until an agreement was reached.

### 2.3. Procedures

The initial step in engaging with the women involved establishing a primary contact point through the association, which then granted approval for the execution of our work. Following this approval, interviews were conducted via video calls at times that were convenient for the women, with all communications facilitated by a designated liaison from the association.

The questionnaires were carried out between 1 December 2021 and 28 February 2022, featuring an open research question that said, “If you have pelvic floor problems, tell me how they affect your daily life?” This encouraged women to describe in their own words their experiences regarding this type of problem and adding further open questions to encourage discussion (Appendix A). The questionnaire was filled out by 160 women over 18 years of age.

The data collection was carried out at the same time the analysis was being conducted. Data analysis was performed in Spanish. During the data analysis, all the authors agreed to point out the same limits of the transcribed texts. The entire text was transcribed into English by a native translator, then back-translated into Spanish by another translator to ensure that the meaning was the same, with the final text validated by the researchers.

### 2.4. Data Analysis

Data were analyzed using inductive qualitative content analysis, a systematic approach that explores variations within data and highlights differences and similarities [21,22,23]. Qualitative content analysis focuses on systematically interpreting and understanding communicative data with the aim of achieving both a condensed and comprehensive description of the phenomenon [17,24].

Haase’s adaptation of Colaizzi’s method was used to analyze the discourses of the participants [25,26,27]. The analysis included reading the discourses several times to understand the meanings conveyed, identifying significant phrases and reformulating them to validate the meanings by consensus in the research team, then identifying and organizing the themes before developing a complete description of those themes [27]. Three researchers participated in the thematic analysis to ensure the consistency of the analysis and the results [17].

During the process, the criteria for methodological rigor of credibility, dependability, confirmability, and transferability were observed [28]. Credibility was achieved by in-depth interviews followed by peer debriefing [27,28,29]. Two coauthors analyzed the transcripts independently by bracketing data on preconceived ideas and strictly following the adapted Colaizzi method described above [27,28,29]. The team then compared and discussed findings until a consensus on the themes and subthemes was achieved [27,28,29]. Confirmability was established by considering variations in participant characteristics and sufficient quotations collected through in-depth interviews [27,28,29]. The audit trail was maintained to ensure all analysis steps could be traced back to the original interviews [27,28,29].

## 3. Results

A total of 160 women with a mean age of 36.2 years participated. The rigorous analysis of the discourses of the 160 participants allowed for four main representative themes to convey the experiences of these women with pelvic floor problems and how their daily lives are affected: “Physical Impacts”, “Emotional and Psychological Impacts”, “Social and Relational Impacts”, and “Sexual Health Impacts”. In addition, twelve subthemes were identified within the four larger themes (Table 1, Table 2, Table 3 and Table 4). The content of each major theme and subtheme is described below and illustrated using quotes from participants in the tables. 

### 3.1. Physical Impact (Table 1)

The study broadly found that participants perceived their physical activity levels to be adversely impacted by pelvic floor disorders, with significant effects extending beyond mere physical limitations to encompass their social engagements as well as their mental and emotional well-being. These individuals reported experiencing pain and a pervasive fear of incontinence during various physical exertions, including, but not limited to, activities such as jumping, laughing, and even sneezing. This apprehension around accidental urine leakage notably diminished their willingness to engage in physical activities, thereby exacerbating the isolation and impacting their quality of life. Within the narratives provided by the participants, three distinct subthemes emerged, offering deeper insights into the multifaceted impact of pelvic floor disorders.

The narratives provided by the participants reveal the pervasive impact of pelvic floor disorders on multiple aspects of their lives, from daily routines to emotional well-being. The emergent themes underscore the importance of comprehensive care strategies that address not only the physical symptoms of PFDs but also the psychological and social challenges faced by those living with these conditions.

#### 3.1.1. Impact on Daily Life

This subtheme encapsulates the profound disruptions caused by pelvic floor disorders in the routine activities of the affected individuals. The fear of urine leakage significantly limited their participation in everyday activities and social interactions, leading to a noticeable decline in their overall lifestyle quality. The limitations imposed by pelvic floor disorders on physical activities also contributed to a sense of loss, affecting individuals’ independence and their ability to engage in leisure activities that were previously enjoyed.

#### 3.1.2. Impact on Emotions

The emotional repercussions of living with pelvic floor disorders were highlighted as a critical concern. Participants spoke of the embarrassment, anxiety, and loss of confidence associated with the possibility of urine leakage in public. This constant worry not only heightened their emotional distress but also led to social withdrawal and isolation, further compounding the psychological impact of their condition. 

#### 3.1.3. Self-Management 

Within pelvic floor disorders, urinary incontinence made many of the participants try to adjust their daily tasks to live with these unwanted symptoms while also modifying their lives to manage their condition by applying the necessary skills to maintain adequate psychosocial functioning. Participants also discussed various self-management strategies they employed to cope with their condition. These strategies included both behavioral modifications, such as planning activities around the availability of restrooms, and physical interventions like pelvic floor exercises aimed at strengthening the muscles and mitigating symptoms.

### 3.2. Emotional and Psychological Impacts (Table 2)

The qualitative analysis of the participants’ testimonies revealed a pervasive sense of discomfort, frustration, and irritation interwoven within the narratives of women dealing with pelvic floor disorders (PFDs). A profound sense of shame associated with their condition was a significant emotional burden, contributing to a marked decrease in self-esteem. Beyond these feelings, some individuals reported experiencing more intense psychological states, including depression, anxiety, and sadness. These heightened emotions were frequently attributed to the debilitating symptoms of fecal and/or urinary incontinence that accompany PFDs, underscoring the profound impact of these conditions on mental health and emotional well-being. Three distinct subthemes emerged: “Discomfort and Insecurity”, “Anxiety and Sadness”, and “Embarrassment”.

Overall, the narratives provided by the women paint a vivid picture of the complex interplay between physical symptoms and emotional responses to PFDs, while the identified subthemes underscore the necessity for holistic care approaches that address both the physical and psychological dimensions of pelvic floor disorders. Such approaches should aim to not only alleviate physical symptoms but to also provide emotional support, destigmatize PFDs, and restore individuals’ confidence and quality of life.

#### 3.2.1. Discomfort and Insecurity

This subtheme captures the physical discomfort and psychological unease that permeate the daily lives of those affected by PFDs. The constant worry over potential incontinence episodes fosters a pervasive sense of insecurity, which often translates into a reluctance to participate in activities that might trigger symptoms, severely limiting individuals’ social and professional lives. Having pelvic floor dysfunction creates a feeling of discomfort in these women, as it becomes necessary to protect themselves, above all, from urine leakage. This means they have to wear a pad, or even a diaper, creating insecurity in their daily lives.

#### 3.2.2. Anxiety and Sadness

The anticipation of incontinence events and the ongoing management of PFD symptoms contribute to a sustained state of anxiety. This emotional strain, coupled with the direct impact of the disorders on individuals’ lifestyles, can precipitate feelings of sadness and hopelessness. The cyclic nature of anxiety and sadness exacerbates the psychological burden of PFDs, creating a significant obstacle to emotional resilience and well-being. These additional feelings appear because the pelvic floor issues make the women feel that they are older than their real ages, causing them to compare themselves with older people, creating a feeling of sadness that is difficult to bear.

#### 3.2.3. Embarrassment

Central to the experiences shared by participants is the acute embarrassment stemming from their symptoms. The social stigma associated with incontinence challenges individuals’ self-image and societal roles, leading to feelings of humiliation and a profound loss of dignity. This embarrassment is not only a source of emotional distress but also a barrier to seeking help and discussing their condition openly, further compounding the challenges faced in managing PFDs. The women feel bad, either due to the continued use of absorbent systems and/or because they think that the people around them will notice, potentially being able to smell the urine in those absorbents.

### 3.3. Social and Relational Impacts (Table 3)

The research findings reveal a pervasive issue among participants: the disruption of continuous sleep due to frequent nocturnal awakenings. This problem is primarily associated with the need to manage urinary incontinence, compelling individuals to either use absorbent products, such as pads or diapers, or to wake multiple times to urinate. The necessity to change these protective aids during the night to avoid remaining in a state of discomfort due to urine wetness is a significant concern. This cycle of interrupted sleep not only compromises the quality of rest but also results in daytime irritability and a general sense of fatigue, attributable to insufficient restful sleep. Two critical subthemes have emerged from the analysis that highlight broader socio-cultural and interpersonal dynamics at play.

#### 3.3.1. Address the Culture of Silence and Shame 

This subtheme reflects a societal issue where discussions around urinary incontinence are often stifled by shame and embarrassment. The stigma attached to incontinence contributes to a culture of silence, preventing individuals from seeking help or sharing their experiences. By challenging this culture and promoting open dialogue, there is the potential to destigmatize urinary incontinence, encouraging individuals to seek and access support and solutions. Education and awareness efforts can play a pivotal role in shifting perceptions, demonstrating that incontinence is a medical issue rather than a matter of personal failing.

#### 3.3.2. The Impacts on Relationships with Partners and Social Interactions 

The repercussions of urinary incontinence extend into the personal lives of those affected, influencing intimate relationships and social engagements. The fear of incontinence episodes during social activities can lead to avoidance behaviors, reducing participation in social gatherings and potentially straining relationships with partners and peers. Intimacy issues may arise that are driven by concerns about the unpredictability of incontinence and its perceived implications for desirability and sexual health. Addressing these impacts requires a compassionate, understanding approach that includes both the individuals experiencing incontinence and their partners, focusing on communication, mutual support, and seeking therapeutic interventions when necessary.

### 3.4. Sexual Health Impact (Table 4)

A subset of the study’s female participants reported experiencing either minimal or no significant symptoms of pelvic floor dysfunction affecting their sexual functions. This specific group of individuals predominantly encountered physical discomfort during sexual activities that occurred in both partnered and solitary scenarios. These findings underscore the multifaceted impact of pelvic floor dysfunction on sexual functions among women. The nuanced understanding of these subthemes highlights the importance of addressing these issues in a comprehensive manner, considering both the physical and psychological dimensions of sexual health. The qualitative analysis of their experiences allowed for the identification of four distinct subthemes that characterize their challenges.

#### 3.4.1. Pain on Penetration and Lack of Sexual Desire

This subtheme encompasses the physical discomfort or pain experienced by some women during the act of penetration. This symptom can significantly hinder sexual activity and contribute to a reluctance or fear of engaging in sexual intercourse. Several participants reported a noticeable decrease in their sexual desire or libido, which they attributed to their pelvic floor dysfunction. This reduction in sexual interest can strain personal relationships and affect overall sexual satisfaction.

#### 3.4.2. Fear of Coital Incontinence 

The fear of experiencing incontinence during coitus emerged as a significant concern for some women. This fear can lead to anxiety about engaging in sexual activities, potentially leading to avoidance behaviors and impacting sexual intimacy. The women expressed a fear of urine leakage during sexual activity, and this caused them to feel embarrassed, resulting in them not wanting to engage in sexual activity with their partner. 

#### 3.4.3. Effects on Arousal Orgasm 

Participants also described challenges related to achieving arousal and orgasm, indicating that pelvic floor dysfunction can interfere with these fundamental aspects of sexual response. This interference can detract from the quality of sexual experiences and personal satisfaction. Finally, a recurring theme in women’s discourses was problems reaching orgasm and a lack of lubrication coupled with less pleasure, leading to these women not wanting to engage in sexual activity, decreasing their quality of life.

## 4. Discussion

The impact of pelvic floor dysfunction on women is complex and affects many aspects of life. This study broadens our understanding of the experience of women with pelvic floor dysfunction and how their lives and the different spheres around them are affected.

The results of this study indicate that women with pelvic floor dysfunction experience a series of problems that decrease their quality of life in different spheres of their lives, such as physical activity, night-time rest, and sexual activity. However,, not all women have these problems and/or do not express it for fear of what others may say, as reported in previous studies [30,31,32].

Some women described pelvic floor disorders as causing urinary incontinence problems during physical activity, night-time rest, and sexual activity [30,32,33], having a negative impact on their personal, social, and physical interactions. This was often attributed to anxiety and stress generated by feelings of discomfort and insecurity, as also described by other authors [34,35]

A feeling of embarrassment and resignation was experienced by the women interviewed regarding moments with their partners or social situations with family and friends resulting from urinary incontinence. Similar concerns have been reported in other studies carried out with women who experienced embarrassment, stress, and low quality of life due to urinary incontinence [36,37,38].

In their discourses, some women reported a culture of silence and shame around the problems derived from pelvic organ dysfunction. Moreover, pain during penetration and a lack of sexual desire caused women to lose interest in sexual activity with their partners, as previously described in the literature [9,14,32,39].

Embarrassment due to incontinence triggers the fear that incontinence will occur during sex. This fear of coital incontinence has the potential to impact all stages of sexual arousal, which is consistently confirmed in the literature, with a desire on the part of women to pretend everything is normal with their partners and hide this type of problem [30,32,33].

Finally, the analysis of the discourse of our participants indicates that the problem that these women have in reaching orgasm due to the lack of lubrication in their vagina most commonly affects their willingness to participate in sexual activity. Furthermore, adverse effects on arousal and orgasm are also observed, indicating that they often changed their practices of sexual intimacy due to the embarrassment or discomfort of their prolapse. Many women reported a complete avoidance of physical intimacy and sexual intercourse, resulting in decreased overall quality of life, as previously reported in other research [30,32,33,40].

### Limitations

The sample was recruited from support groups on social networks, which could mean that these women have actively sought more information on and support for their pelvic floor disorders. The use of qualitative methods to explore pelvic floor dysfunction in women is the main strength of this study, as it provides the direct perspective of those women who experience it, explaining in their own words the symptoms that really bother them. Women are often overlooked when looking at purely quantitative data; hence, our approach may allow individual management strategies to be tailored to the particular requirements of each woman.

## 5. Conclusions

Our research has conclusively demonstrated that disorders of the pelvic floor precipitate a multitude of complications in women, significantly diminishing their overall quality of life. These issues extend across various aspects of daily living, including disruptions to sleep patterns, limitations of physical activity, and notable impediments to sexual function. A noteworthy finding from our study is the tendency among affected women to under-report these challenges, often due to feelings of fear or embarrassment. This reticence leads to a concerted effort to maintain an outward appearance of normalcy, concealing their struggles not only from their partners but also, in many cases, from healthcare professionals.

The implications of our findings underscore the pressing need for proactive measures by public health entities. These organizations play a crucial role in both the early detection of pelvic floor disorders and the allocation of resources necessary for effective intervention. Early visibility and intervention are paramount, as they can significantly mitigate the long-term impacts of these disorders on women’s health and well-being.

Moreover, our study advocates for a broader societal awareness regarding pelvic floor dysfunction. Educating the general population about the importance of pelvic health and the potential consequences of ignoring or delaying treatment for these disorders is essential. This awareness should be aimed not only at potential patients but also at their partners and families, healthcare providers, and policymakers. Enhancing understanding and reducing stigma can encourage more women to seek help early, improving outcomes and reducing the overall burden of pelvic floor disorders.

In conclusion, our research highlights the multifaceted impact of pelvic floor disorders on women’s lives and emphasizes the necessity for increased visibility, awareness, and proactive health measures. By addressing these issues comprehensively, we can improve the quality of life for affected individuals and contribute to the broader goal of enhancing women’s health outcomes globally.

## Figures and Tables

**Table 1:** Theme, Subthemes and Verbalizations.

Theme	Subthemes	Verbatims
***Physical Impacts***	Impact on daily life	“Urinary incontinence makes it impossible for me to do satisfactory physical activity due to the pressure I feel in my parts.” “Urine leaks when I make an effort, whether running or jumping. I can’t perform all the exercises that I would like to.” “I have mild urinary incontinence, if I overexert or sneeze or laugh, some urine always leaks.” “I have incontinence when carrying weight, climbing stairs, or walking fast. I have to go to the bathroom frequently and stop whatever activity I am doing.”
	Impact on emotions	“Discomfort and the need to constantly use a pad makes me feel helpless because I depend exclusively on whether I am wearing a pad, or else I am going to urinate on myself.” “I have no problems, but I am afraid that when I do physical activity I will have problems with urinary incontinence.” “I have urinary incontinence that limits me in some activities. It is uncomfortable and embarrassing.” “I feel powerless because there are times when I pee a lot, and I don’t have time to get to the bathroom without leaking, which sometimes means I don’t leave my house as I´m scared this will happen to me outside and it will stain the clothes.” “I don’t feel ready to exercise for fear of worsening my prolapse.” “I feel like I’m in a bad mood all day due to the discomfort and discomfort in the pelvic and vaginal area, a feeling of weight due to prolapse, itching, and the feeling that my bladder is not emptying...” “On an emotional level, it makes me think a lot about what happens to me and its consequences with my partner and my perspective as a woman.”
	Self-management	“I have urine leaks that force me to do physical activity with incontinence pads and go to the bathroom frequently if I don’t want to urinate on myself.” “I need pads continuously for urine losses.” “I have to always use pads due to urine losses with non-aerobic exertion and impact exercises.” “I have reduced my physical activity and the sports that I like since I can’t do them because of urine leaks.” “Incontinence prevents me from running. I need to know that there will be a bathroom in any long-term activity.”

**Table 2:** Theme, Subthemes and Verbalizations.

Theme	Subthemes	Verbatims
***Emotional and Psychological Impacts***	Discomfort and insecurity	“I always have to wear a pad. I don’t want to do physical exercise because of embarrassment and insecurity, when I work it’s the only time I allow myself to make efforts because it’s necessary; otherwise I wouldn’t do it, I feel frustrated with my life.” “I don’t go far from home, I always need a bathroom nearby and pads or absorbent panties, this causes me to be irritated and uncomfortable all day since I can’t carry out my life as I would like” “Having to be careful not to urinate on myself is annoying and causes to feel insecure in my daily life.” “It causes me discomfort and concern when doing sports.” “Discomfort, insecurity, continually going to the bathroom… Above all, being away from home for a long time and meeting my friends.”“I don’t feel safe, it’s very unpleasant. Whenever I go out I have to go to the bathroom urgently many times.”
	Anxiety and sadness	“I don’t use a pad because I don’t want to, but it would be necessary, it’s hard for me to get up to urinate, I can’t make any physical effort, all I do is sew. Sometimes I have time to get to the bathroom and sometimes not. I have a sofa cover to be able to wash it, and so I force myself to get up to the bathroom, and if I don’t get there I stain it. This situation causes me anxiety.” “Having to wear pads affects me a lot on a psychological level, continuously creating anxiety.” “I suffer from anticipatory anxiety in front of social encounters, I get sick just thinking about it.” “In sexual enjoyment, I have lost desire due to the discomfort it generates. Also in the freedom to walk, the discouragement and social isolation that I feel, it makes my life very complex.” “Having to think about wearing pads or having a bathroom nearby makes me have a worse mood, I also notice pressure in the pelvic area. I feel like I’m older than I am.”
	Embarrassment	“Having pelvic problems causes me anxiety, shame, and depression. I consider myself young, and I do not want to wear a pad and have to constantly think about the bathroom.” “It limits some activities; it is embarrassing, and this feeling makes me sad.” “The smell that the pee gives off makes me anxious and ashamed because the people around me can smell it.” “I urinate frequently and have urine leaks; I am embarrassed to have to always keep an eye on carrying things in my bag or having a bathroom nearby.” “It embarrasses me and emotionally bothers me to have urine leaks, which affects my day-to-day.” “It’s annoying to have to think about carrying a pad in your bag and also have the embarrassment of always being in the bathroom when you go out with friends.”

**Table 3:** Theme, Subthemes and Verbalizations.

Theme	Subthemes	Verbatims
***Social and Relational Impacts***	Address the culture of silence and shame	“I feel pelvic pain at night, which makes me unable to sleep properly.” “At night, I have to repeatedly go to the bathroom, waking up, and not letting me rest.” “I have to get up to the bathroom several times during the night, and I don’t sleep well.” “I usually get up to urinate about three times a night. If I am nervous, this increases until I have to go every half hour.” “Getting up to the bathroom a couple of times at night makes me unable to rest.” “Not holding urine makes me lose sleep, and having to get out of bed to go to the bathroom makes me not rest.” “I don’t sleep well almost every night because I have to get up to urinate, because otherwise my abdominal pressure increases.” “Not holding urine all night or having to urinate several times makes it very difficult for me to fall asleep and stay asleep.”
	The impacts on relationships with partners and social interactions	“I wear an absorbent at night (panties, diaper), I go to the bathroom a lot, and sometimes I do not even get there, disturbing my night’s rest.” “It interrupts my sleep to get up to go to the bathroom. You sleep with fear of leaking urine.” “I am afraid of falling into a deep sleep and peeing during the night while dreaming that I am in the bathroom.” “You are not calm, because you are afraid of leaking urine during sleep.”

**Table 4:** Theme, Subthemes and Verbalizations.

Theme	Subthemes	Verbatims
***Sexual health impact***	Pain on penetration	“Sometimes I have pain during penetration with certain positions.” “I feel pain, I have a contracture. I’m afraid of penetration.” “I have pain and difficulty lubricating.” “I feel pain, scared of penetration. It bothers me even when my partner brings up the subject. No sexual appetite, even displeasure.” “I have not had penetrative sexual activity due to lack of interest and also for fear of pain.”
	Lack of sexual desire	“Lack of sexual desire. Dryness, burning, pain. Lack of lubrication and pleasure. Low desire and finishing. Lack of interest in the relationship. We hardly have any sex.” “Air enters my vagina when I walk, it is uncomfortable. I cannot use a menstrual cup because it drops out, in some sexual positions I don’t feel anything and I feel very open. All this affects the sexual quality with my partner.”
	*Fear of coital incontinence*	“Because of the fear of urine leaking, pain, and anal sex, I do not like it. It makes me want to pee.” “During sex, urine leaks and I have to take a break. I feel embarrassed.” “I’m terrified that I might poop or pee while we’re at it. I almost always tell my partner no because of that fear. I get tense, it hurts, but there are times when I have to accept, even if I don’t have a good time.” “It affects my sexual activity and therefore my emotional state, I feel ashamed in the relationship with my partner, I feel bad about my body. All because of urine loss...”
	*Effects on arousal orgasm*	“I have difficulty lubricating and having an orgasm.” “I have less arousal and less pleasure during orgasm.” “There are positions in penetration that bother me a bit in the uterus, and I have less sensitivity than before being a mother. It is difficult for me to reach orgasm with penetration, I only have orgasms with clitoral stimulation.”

## Data Availability

Data is available on responsible request to the corresponding author.

## Appendix A. Interview Questions

- Explain what it is like for you to live with a pelvic floor disorder (problem)?
- If you have pelvic floor problems (prolapse, urinary incontinence and/or fecal incontinence), does it affect your quality of life? Explain in what way it affects you.
- If you have pelvic floor problems (prolapse, urinary incontinence and/or fecal incontinence), does it affect you when carrying out physical activity? Explain in what way it affects you.
- If you have pelvic floor problems (prolapse, urinary incontinence and/or fecal incontinence), does it affect your quality of sleep? Explain in what way it affects you.
- If you have pelvic floor problems (prolapse, urinary incontinence and/or fecal incontinence), does it affect your sexual functioning? Explain in what way it affects you.
